# Insights into transcription termination of Hfq-binding sRNAs of *Escherichia coli* and characterization of readthrough products

**DOI:** 10.1261/rna.051870.115

**Published:** 2015-08

**Authors:** Teppei Morita, Masaki Ueda, Kento Kubo, Hiroji Aiba

**Affiliations:** Faculty of Pharmaceutical Sciences, Suzuka University of Medical Sciences, Suzuka, Mie 513-0816, Japan

**Keywords:** Hfq, bacterial sRNA, Rho-independent terminator, transcriptional readthrough, termination and stress

## Abstract

The genes encoding Hfq-dependent sRNAs possess a typical Rho-independent transcription terminator. Here, we have studied the molecular events occurring at Rho-independent terminators of sRNA genes, focusing on two well-characterized Hfq-binding sRNAs, SgrS and RyhB. We constructed several hybrid genes in which the DNA sequence corresponding to a strong Rho-independent terminator was placed just downstream from the Rho-independent terminators of sRNA genes. By using this system, we demonstrate that transcripts frequently read through the Rho-independent terminators of *sgrS* and *ryhB* in normally growing cells. We show that Hfq does not affect the transcriptional readthrough event itself. We also find that the readthrough products no longer bind to Hfq in vivo. We have developed a competition assay based on a biotin–streptavidin system to analyze the interaction of Hfq and a particular RNA molecule in vitro. By using this method, we verify that the 3′-extended form of SgrS does not bind to Hfq in vitro. Finally, we demonstrate that transcription termination is significantly enhanced under stress conditions where transcription initiation of sRNA genes on the chromosome is induced. We conclude that the production of sRNAs is regulated not only at the step of transcription initiation but also at the step of transcription termination. The mechanism by which transcription termination is enhanced under stress conditions remains to be understood.

## INTRODUCTION

Major regulatory small RNAs (sRNAs) in bacteria bind to the RNA chaperone Hfq and regulate the translation and the stability of target mRNAs through base-pairing by the help of Hfq (Waters and Storz 2009; Gottesman and Storz 2010; Vogel and Luisi 2011). The transcription initiation of sRNA genes is induced under specific physiological and/or stress conditions because the promoter of an individual sRNA gene is under the control of a specific transcription factor that is modulated by respective stress conditions. For example, *Escherichia coli* SgrS is induced in response to glucose-phosphate stress such as accumulation of glucose-6-phosphate (Vanderpool and Gottesman 2004). The induced SgrS sRNA pairs with the target mRNAs to either down- or up-regulate their expression (Vanderpool and Gottesman 2004; Morita et al. 2005; Papenfort et al. 2013). A transcription factor called SgrR is activated, by unknown mechanisms, under the glucose-phosphate stress to stimulate the transcription from the *sgrS* promoter by RNA polymerase (Vanderpool and Gottesman 2007). Another well-characterized Hfq-binding sRNA, RyhB, is induced in response to Fe^2+^ depletion to regulate the expression of several mRNAs encoding Fe-binding proteins through base-pairing mechanism (Masse and Gottesman 2002; Masse et al. 2003). In this case, Fur, a repressor protein for the *ryhB* promoter, is inactivated by the Fe^2+^ depletion, leading to expression of RyhB (Masse and Gottesman 2002).

The expression of sRNA genes might be regulated also at the step of transcription termination. Genes encoding Hfq-dependent sRNAs are often located in intergenic regions as a single gene (Waters and Storz 2009) while some sRNA genes form operons along with other genes and/or are embedded in the 3′ region of protein-coding genes (Sun and Vanderpool 2011; Chao et al. 2012; Guo et al. 2014). In either case, the sRNA genes possess a typical Rho-independent transcription terminator that is likely to specify the 3′ends of most sRNAs. The hallmark features of Rho-independent terminators, also called factor-independent or intrinsic terminators, are a short GC-rich inverted repeat sequence followed by a run of T residues on the nontemplate strand (d'Aubenton Carafa et al. 1990; Reynolds et al. 1992). When RNA polymerase transcribes the inverted repeat sequence of the terminator, the resulting RNA forms a stem–loop (hairpin) structure that is believed to trigger transcription termination within transcribed U stretches by disrupting the elongation complex due to weak A–U base pairs (Rosenberg and Court 1979; Platt 1986; Yager and von Hippel 1991). We noticed previously that the Rho-independent terminators of sRNA genes possess longer T residue stretches (more than seven) on the nontemplate strand (Otaka et al. 2011) than most other terminators which can vary depending on terminators, but usually less than six (d'Aubenton Carafa et al. 1990). We showed recently that the long polyU tail and hairpin of sRNAs corresponding to their Rho-independent terminator along with a preceding internal U-rich sequence consist of the functional Hfq-binding module of sRNAs (Ishikawa et al. 2012). This implies that the consecutive terminator A residues on the template strand must be efficiently transcribed to generate functional sRNAs. Thus, insights into transcription termination and its regulation will be important to understand how functional sRNAs are generated in cells. To date, however, molecular events at Rho-independent terminators of sRNA genes have been poorly studied. For example, it is largely unknown how frequently transcription termination or readthrough occurs beyond sRNA genes. Because the terminator hairpin and the long polyU tail are essential elements for the Hfq-binding module, another interesting question is whether Hfq affects the transcription termination/readthrough at Rho-independent terminators of sRNA genes. Furthermore, it is not known whether the 3′-extended forms of sRNAs generated by transcriptional readthrough at sRNA terminators still retain the Hfq-binding ability although an early mutational study suggests that the 3′ extension of DsrA sRNA lead to the loss of its regulatory function (Sledjeski and Gottesman 1995).

The aim of this study is to address these questions, focusing on two well-characterized Hfq-binding sRNAs, SgrS and RyhB. By using a “double terminator system” (Abe et al. 1999), we demonstrated that transcriptional readthrough frequently occurs at Rho-independent terminators of sRNA genes and that the readthrough is not affected by Hfq. In addition, we found that the readthrough products, the 3′-extended forms of sRNAs, no longer bind to Hfq both in vivo and in vitro. Finally, we found that transcription termination is markedly enhanced under stress conditions in which the transcription of sRNA genes on the chromosome is induced in cells. The biological relevance of this finding will be discussed.

## RESULTS

### Double terminator system

**FIGURE 1.**
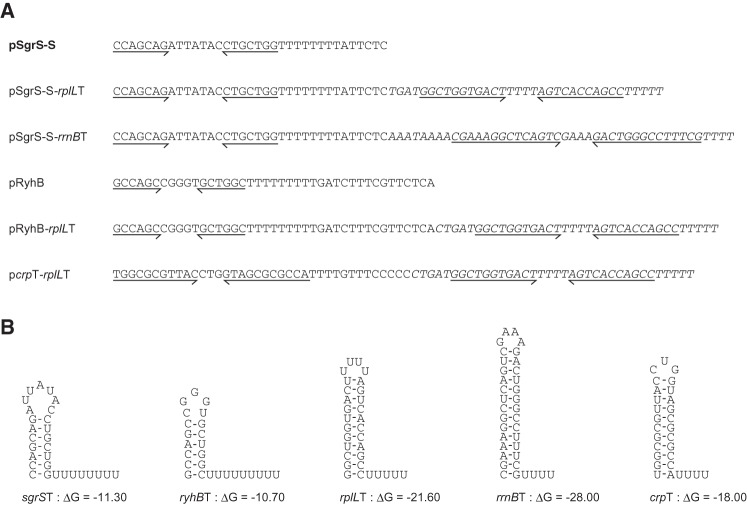
(*A*) DNA sequences around Rho-independent terminators of genes used in this study. The sequences corresponding to Rho-independent terminators of *sgrS* and *ryhB* are shown as regular letters, whereas the terminator sequences derived from *rplL* and *rrnB* are shown as italic letters. The inverted repeat sequences of Rho-independent terminators are shown by horizontal arrows. (*B*) The predicted secondary structures of transcripts corresponding to Rho-independent terminators. The free energies of terminator hairpin structures were determined according to the Mfold program (Zuker 2003).

The efficiency of termination at Rho-independent terminators is known to vary widely among individual terminators (Reynolds et al. 1992). Thus, elongating RNA polymerases pass through different terminator structures with different frequencies, leading to transcription of the downstream sequences. It is generally difficult to evaluate the efficiency of termination by analyzing RNA transcripts directly by Northern blotting because readthrough products are often heterogeneous in size and/or perhaps unstable. We have used a “double terminator system” (Abe et al. 1999) in which a strong Rho-independent terminator derived from *rrnB* (*rrnB*T1) is placed just downstream from a Rho-independent terminator to be tested. The downstream strong terminator efficiently traps the readthrough RNA that is made beyond the test terminator, resulting in stable and distinct longer RNA molecules. To know how frequently transcription termination occurs at Rho-independent terminators of sRNA genes, we constructed hybrid genes on a plasmid in which a strong Rho-independent terminator of *rplL* (Post et al. 1979) or *rrnB* (Brosius et al. 1981) was placed just downstream from the Rho-independent terminators of *sgrS* and *ryhB* genes encoding SgrS and RyhB sRNAs, respectively. The DNA sequences around the terminators of the genes used along with the predicted RNA secondary structures of transcripts corresponding to the Rho-independent terminators are shown in Figure 1.

### Evaluation of transcriptional readthrough at the *sgrS* terminator

**FIGURE 2.**
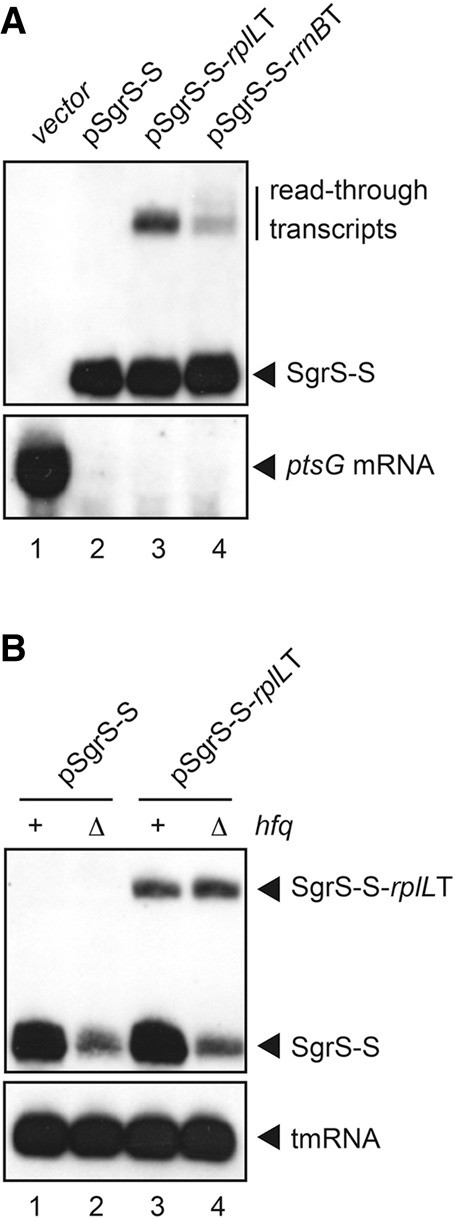
(*A*) Properties of transcripts generated from *sgrS-S* and hybrid genes. TM542 (Δ*sgrS hfq*^+^) cells harboring indicated plasmids were grown in LB medium in the presence of 0.2% arabinose. Total RNAs were prepared and 10 μg of RNA samples were subjected to Northern blotting using SgrS-S probe and *ptsG* probe. (*B*) Effect of Hfq on expression of SgrS-S and SgrS-S*-rplL*T. TM542 (Δ*sgrS hfq*^+^) and TM772 (Δ*sgrS* Δ*hfq*) cells harboring indicated plasmids were grown in LB medium in the presence of 0.2% arabinose. Total RNAs were prepared and 10 and 0.25 μg of RNA samples were subjected to Northern blotting using the SgrS-S probe and tmRNA probe, respectively.

We first focused on the *sgrS* terminator. Plasmid pSgrS-S carries the *sgrS-S* encoding SgrS-S that corresponds to the 3′ portion of SgrS consisting of the minimal base-pairing region and the Hfq-binding module including the Rho-independent terminator sequence (Otaka et al. 2011; Ishikawa et al. 2012). Plasmids pSgrS-S-*rrnB*T1 and pSgrS-S-*rplL*T carry the hybrid gene *sgrS-S*-*rrnB*T1 and *sgrS-S*-*rplL*T, respectively. The *sgrS-S* gene is under the control of an arabinose-inducible promoter in these plasmids. We first examined transcription termination/readthrough at the *sgrS* terminator in *hfq*^+^ background by Northern analysis. Plasmids described above were introduced into *hfq*^+^ cells. As a control, cells were also transformed with the vector plasmid pAraX (Ishikawa et al. 2012). Cells were grown in the presence of arabinose and expression of sRNAs was analyzed by Northern blotting. The *sgrS-S* gene generates ∼60 nt SgrS-S RNA (Fig. 2A, lane 2). As expected, two hybrid genes, *sgrS-S*-*rrnB*T1 and *sgrS-S-rplL*T, also produce the 60 nt SgrS-S (Fig. 2A, lanes 3,4). The expression levels of SgrS-S were essentially identical among these three genes (*sgrS-S*, *sgrS-S*-*rrnB*T1, and *sgrS-S*-*rplL*T). Thus, transcription termination at the *sgrS* terminator is not affected by the downstream second terminator *rrnB*T1 or *rplL*T. We also confirmed that the target *ptsG* mRNA was dramatically destabilized when SgrS-S was expressed from any of the three genes (Fig. 2A, lower panel), indicating that SgrS-S generated from the hybrid genes is functional to down-regulate the *ptsG* mRNA. As expected, longer RNA bands corresponding to SgrS-S-*rrnB*T1 and SgrS-S-*rplL*T are produced in addition to SgrS-S band in cells harboring pSgrS-S-*rrnB*T1 or pSgrS-S-*rplL*T (Fig. 2A, lanes 3,4). These bands can be produced mainly because transcripts that passed through the *sgrS* terminator terminate at the downstream *rrnB*T1 or *rplL*T terminator. It is reported that the termination efficiency of *rrnB*T1 in vivo is >90% (Brosius et al. 1981). The *rplL*T is expected to be also strong because of the high stability of its terminator hairpin structure (Fig. 1B). Indeed, the band corresponding to SgrS-S-*rplL*T is much more distinct than that of SgrS-S-*rrnB*T1. Hereafter, we have used the *rplL*T as the downstream terminator to trap the readthrough products.

**FIGURE 3.**
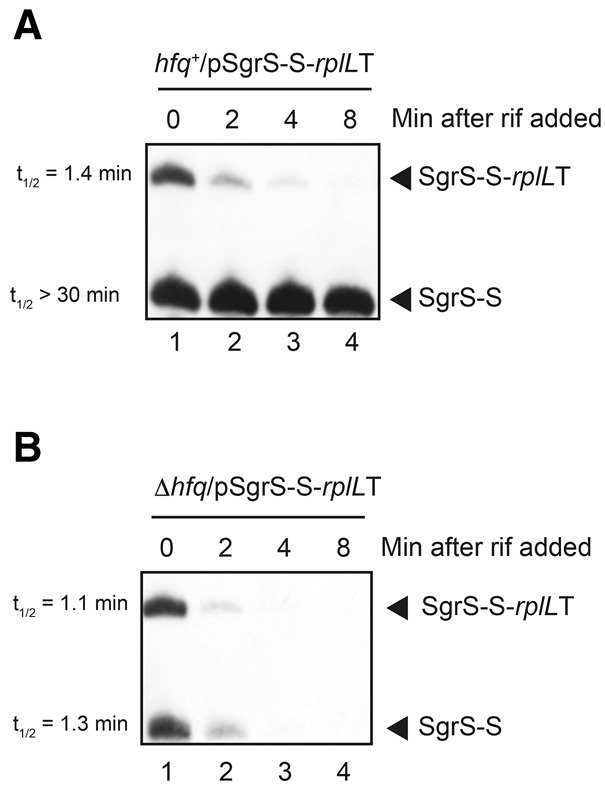
Stability of SgrS-S and SgrS-S*-rplL*T. (*A*)TM542 (Δ*sgrS hfq*^+^) and (*B*) TM772 (Δ*sgrS* Δ*hfq*) cells harboring pSgrS-S-*rplL*T were grown in LB medium in the presence of 0.2% arabinose to *A*_600_ = 0.6. Rifampicin (250 μg/mL) was added and the incubation was continued. Total RNAs were prepared at the indicated time after the addition of rifampicin. The RNA samples (10 μg) were subjected to Northern blotting using the SgrS-S probe.

The presence of Hfq in cells may affect differentially the accumulation of terminated SgrS-S and elongated SgrS-S-*rplL*T because SgrS-S is stabilized by Hfq binding (Otaka et al. 2011), while it is not sure at this moment whether the stability of SgrS-S-*rplL*T is affected by Hfq. We examined the accumulation of terminated and elongated products in both *hfq*^−^ and *hfq*^+^ backgrounds. As expected, the abundance of SgrS-S generated from either pSgrS-S or pSgrS-S-*rplL*T is markedly reduced in the *hfq*^−^ background as compared with the *hfq*^+^ background (Fig. 2B, lanes 2,4). In contrast, the abundance of SgrS-S-*rplL*T band generated from pSgrS-S-*rplL*T is not reduced in the *hfq*^−^ background (Fig. 2B, lane 4), suggesting that SgrS-S-*rplL*T is not stabilized by Hfq. Thus, it is appropriate to compare the relative abundance of SgrS-S-*rplL*T and SgrS-S bands in the *hfq*^−^ background rather than in the *hfq*^+^ background to evaluate the efficiency of termination/readthrough at the *sgrS* terminator. Interestingly, the abundance of the SgrS-S-*rplL*T band is almost identical with that of the SgrS-S band (Fig. 2B, lane 4). This implies that ∼50% of elongating RNA polymerase reads through the *sgrS* terminator if it is assumed that SgrS-S-*rplL*T and SgrS-S are similar in stability in the *hfq*^−^ background. In fact, we show that this is the case (Fig. 3B).

### The readthrough products are not stabilized by Hfq

The readthrough product (SgrS-S-*rplL*T) contains the sequence corresponding to the Hfq-binding module (Ishikawa et al. 2012) although the polyU stretch is located internal in the RNA molecule rather than at the 3′ end. An interesting question is if SgrS-S-*rplL*T still retains the Hfq-binding ability. The observation that the presence of Hfq does not enhance the abundance of SgrS-S-*rplL*T (Fig. 2B) suggests that SgrS-S-*rplL*T is not stabilized much by Hfq whereas SgrS-S is. To test this, we evaluated the stability of terminated and elongated forms of SgrS-S in both *hfq*^−^ and *hfq*^+^ backgrounds. Cells harboring pSgrS-S-*rplL*T were grown to mid-log phase in the presence of arabinose and rifampicin was added to prevent further initiation of transcription. RNAs were isolated at various times after the addition of rifampicin, and these RNA samples were subjected to Northern blotting. As shown in Figure 3B, the half-lives (*t*_(1/2)_) of SgrS-S and SgrS-S-*rplL*T RNAs are estimated to be 1.3 and 1.1 min, respectively, in *hfq*^−^ cells. Thus, both SgrS-S and SgrS-S-*rplL*T are highly unstable in the absence of Hfq. On the other hands, SgrS-S is dramatically stabilized in *hfq*^+^ cells (*t*_[1/2]_ > 30 min) while SgrS-S-*rplL*T is still unstable in *hfq*^+^ cells (Fig. 3A).

### Analysis of the *ryhB* terminator

**FIGURE 4.**
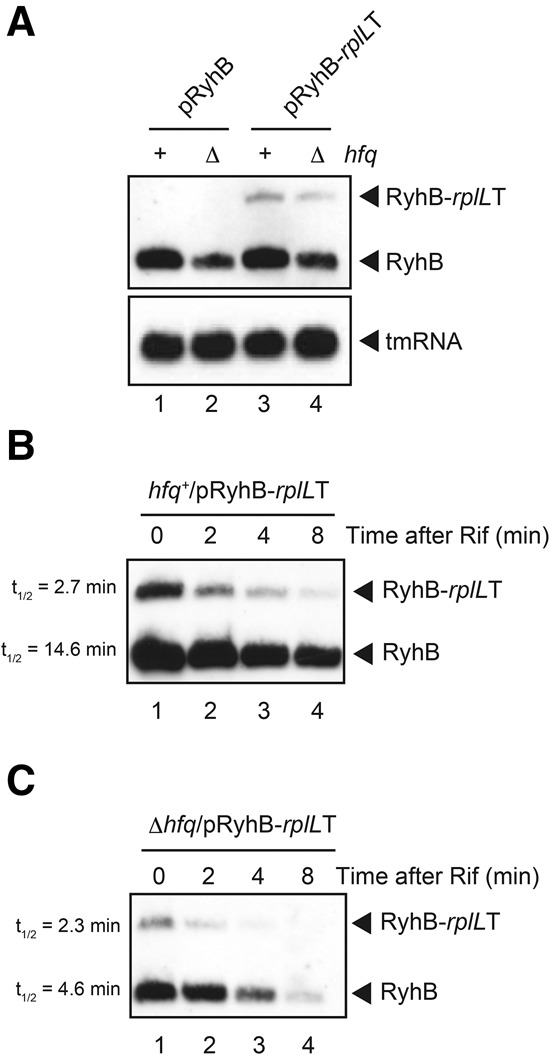
(*A*) Transcripts generated from *ryhB* and *ryhB-rplLT* genes. TM635 (Δ*ryhB hfq*^+^) and TM820 (Δ*ryhB* Δ*hfq*) cells harboring indicated plasmids were grown in LB medium in the presence of 1.0% arabinose. Total RNAs were prepared and 4 and 0.25 μg of RNA samples were subjected to Northern blotting using RyhB probe and tmRNA probe, respectively. (*B*,*C*) Stability of RyhB and RyhB*-rplL*T. TM635 (Δ*ryhB hfq*^+^) (*B*) and TM820 (Δ*ryhB* Δ*hfq*) (*C*) cells harboring pRyhB-*rplL*T were grown in LB medium in the presence of 1.0% arabinose to *A*_600_ = 0.6. Rifampicin (250 μg/mL) was added and the incubation was continued. Total RNAs were prepared at the indicated time after the addition of rifampicin. The RNA samples (10 μg) were subjected to Northern blotting using the RyhB probe.

We also examined transcriptional readthrough at the Rho-independent terminator of the *ryhB* gene encoding another well-characterized Hfq-binding sRNA, RyhB (Masse and Gottesman 2002) with the double terminator system. Plasmids pRyhB and pRyhB-*rplL*T carry the *ryhB* and *ryhB-rplLT* genes, respectively. RNA transcripts from these plasmids in both *hfq*^−^ and *hfq*^+^ cells were analyzed by Northern blotting. The *ryhB* gene produces 95 nt RyhB while the hybrid *ryhB*-*rplL*T gene produces two RNAs, RyhB plus a longer RNA corresponding to RyhB-*rplL*T (Fig. 4A). Based on the ratio of the RyhB-*rplL*T band to the terminated RyhB band, the efficiency of transcription termination at the *ryhB* terminator is estimated to be ∼80% in the *hfq*^−^ background (Fig. 4A, lane 4). The abundance of RyhB generated from both pRyhB and pRyhB-*rplL*T in *hfq*^−^ cells is significantly reduced compared with the *hfq*^+^ background while the abundance of RyhB-*rplL*T is moderately affected by the *hfq*^−^ background (20%–30% less in *hfq*^−^ cells).

We also evaluated the stability of terminated and elongated forms of RyhB in both *hfq*^−^ and *hfq*^+^ backgrounds. Cells harboring pRyhB-*rplL*T were grown to mid-log phase in the presence of arabinose and rifampicin was added to prevent further initiation of transcription. RNAs were isolated at various times after the addition of rifampicin, and these RNA samples were subjected to Northern blotting (Fig. 4B,C). The half-lives of RyhB and RyhB-*rplL*T in the *hfq*^−^ background were estimated to be 4.6 and 2.3 min, respectively (Fig. 4C). It is interesting to note that RyhB is relatively stable compared with SgrS in the *hfq*^−^ background. As expected, RyhB is significantly stabilized (*t*_(1/2)_ = 14.6 min) in *hfq*^+^ cells (Fig. 4B) although the effect of Hfq on RyhB stabilization is not as great as on SgrS-S. RyhB-*rplL*T, like SgrS-S-*rplL*T, is only moderately stabilized in *hfq*^+^ cells.

### Hfq does not bind to the 3′-extended forms of SgrS and RyhB

**FIGURE 5.**
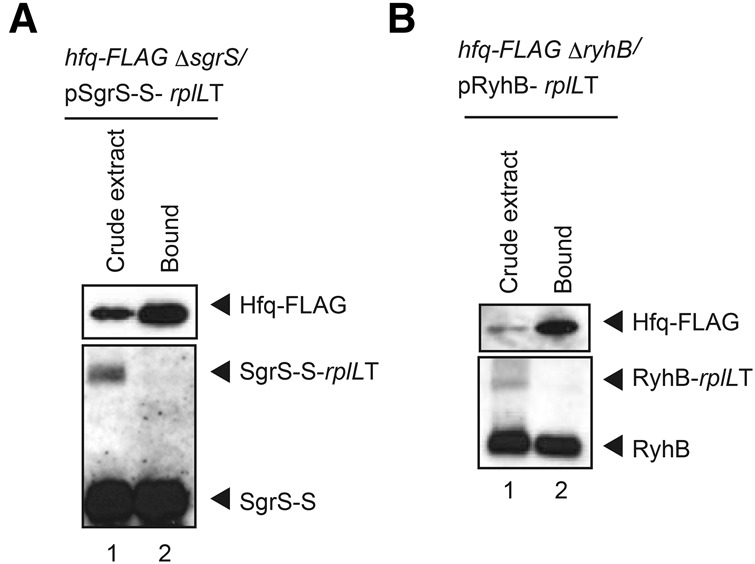
In vivo binding of Hfq to terminated sRNAs and readthrough products. Crude extracts were prepared from TM803 (Δ*sgrS hfq-Flag*) cells harboring pSgrS-S-*rplL*T (*A*) and from TM822 (Δ*ryhB hfq-Flag*) cells harboring pRyhB-rplLT (*B*). Crude extracts were subjected to the pull-down assay using anti-Flag agarose as described in Materials and Methods. Crude extract (CE; 10 μL) and bound fraction (B; 2 μL) were analyzed by Western blotting using anti-Flag antibodies. For analysis of RNAs, crude extract (CE; 10 μL) and bound fraction (B; 2 μL) were treated with phenol and subjected to Northern blotting using the SgrS-S (*A*) or RyhB (*B*) probe.

We showed above that the readthrough products, SgrS-S-*rplL*T and RyhB-*rplL*T, are only moderately stabilized by Hfq. This strongly suggests that the 3′ extension of SgrS and RyhB impairs the Hfq-binding ability of these extended sRNAs. To test this directly, we performed a pull-down assay using cells expressing SgrS-S-*rplL*T along with SgrS-S, and Hfq-Flag. Cell extracts were incubated with anti-Flag M2-agarose beads. Proteins bound to the agarose beads were analyzed by Western blotting using anti-Flag antibodies. The affinity-purified Hfq-Flag was treated with phenol and subjected to Northern blotting. As shown in Figure 5A, SgrS but not SgrS-S-*rplL*T co-purified with Hfq-Flag. We also performed a pull-down assay using cells expressing RyhB-*rplL*T along with RyhB and Hfq-Flag. Again, the terminated RyhB but not the elongated RyhB-*rplL*T co-purified with Hfq-Flag (Fig. 5B). These results indicate that the 3′ extention surely impairs the Hfq-binding ability of sRNAs in vivo.

**FIGURE 6.**
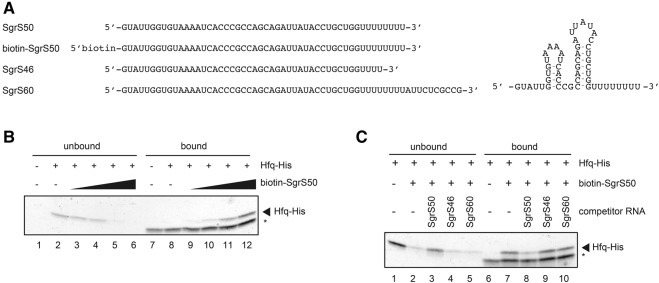
Binding of Hfq to synthetic RNAs in vitro. (*A*) Synthetic RNAs used and their nucleotide sequences. SgrS50 is a 50 nt RNA corresponding to the nucleotides 178–227 of SgrS that is essentially identical with the Hfq-binding module of SgrS (nucleotides 179–227). Biotin-SgrS50 is a derivative of SgrS50 in which the 5′ end was biotinylated. SgrS46 is a derivative of SgrS50 lacking the last 4U of 3′ end. SgrS60 is a derivative of SgrS50 in which the 10 nt sequence corresponding to the readthrough transcript is extended. The predicted secondary structure of SgrS50 is shown on the *right*. (*B*) Interaction between biotin-SgrS50 and Hfq. Streptavidin magnetic beads and Hfq-His_6_ (66.7 nM) were gently shaken with increasing amounts of biotin-SgrS50 in 15 μL of binding buffer for 10 min at 37°C. The following amounts of biotin-SgrS50: lanes *3*,*9*, 8.33 nM; lanes *4*,*10*, 16.7 nM; lanes *5*,*11*, 33.3 nM; lanes *6*,*12*, 66.7 nM. Unbound supernatant and bound precipitate were analyzed by SDS-PAGE and visualized by silver staining. The asterisks (*) indicate the protein (streptavidin) released from the beads during sample heating. (*C*) Effects of SgrS-S variants on the interaction between biotin-SgrS50 and Hfq. Streptavidin magnetic beads, biotin-SgrS50 (33.3 nM) and Hfq-His_6_ (66.7 nM) were gently shaken with indicated RNAs (667 nM) in 15 μL of binding buffer for 10 min at 37°C. Unbound supernatant and bound precipitate were analyzed by SDS-PAGE and visualized by silver staining. The asterisks indicate the protein (streptavidin) released from the beads during sample heating.

We also tested the effect of 3′ extension of SgrS on Hfq binding in vitro by using several synthetic RNAs shown in Figure 6A. SgrS50 contains the entire sequence corresponding to the Hfq-binding module of SgrS including eight consecutive uridine residues at the 3′ end (Otaka et al. 2011; Ishikawa et al. 2012). The predicted secondary structure of SgrS50 is shown on the right of Figure 6A. Biotin-SgrS50 is a derivative of SgrS50 in which the 5′ end of SgrS50 is biotinylated. Both SgrS50 and biotin-SgrS50 are expected to retain the ability to bind Hfq. SgrS46 is a derivative of SgrS50 in which the polyU tail is shortened to 4U residues and therefore is expected to lose the Hfq-binding ability (Otaka et al. 2011). SgrS60 is a derivative of SgrS50 in which 10 bases are extended after the polyU stretch. First, Hfq-His_6_ was incubated with streptavidin-conjugated magnet beads either with or without biotin-SgrS50. Hfq bound and unbound to the magnetic beads was analyzed by SDS-PAGE/silver staining. When Hfq was incubated with the beads in the absence of biotin-SgrS50, Hfq was found only in the unbound fraction (Fig. 6B, lanes 2,8), indicating that Hfq-His_6_ itself does not bind to the beads. On the other hand, Hfq in the unbound and bound fractions decreased and increased, respectively, with increasing amounts of biotin-SgrS50 (Fig. 6B, lanes 3–6, and lanes 9–12). These results imply that Hfq binds stably to biotin-SgrS50 that tightly interacts with streptavidin-conjugated magnet beads. Then, we examined the effect of excess amounts of SgrS50, SgrS46, and SgrS60 on the interaction between Hfq and biotin-SgrS50. When Hfq was incubated with the beads along with biotin-SgrS50 and excess amounts of SgrS50, Hfq was found predominantly in the unbound fraction (Fig. 6C, lanes 3,8). This implies that Hfq binding to biotin-SgrS50 was inhibited because SgrS50 competes well with biotin-SgrS50 regarding Hfq binding. On the other hand, Hfq was found predominantly in the bound fraction when Hfq was incubated with the beads along with biotin-SgrS50 and excess amounts of SgrS46 (Fig. 6C, lanes 4,9). Thus, SgrS46 affects little the interaction between biotin-SgrS50 and Hfq, indicating that SgrS46 essentially does not bind to Hfq as expected. Then, we performed the same competition assay by using excess amounts of SgrS60. This 3′-extended form of SgrS also did not affect the binding of Hfq to biotin-SgrS50 (Fig. 6C, lanes 5,10), indicating that 3′ extension of SgrS also impairs the Hfq-binding ability of SgrS. These results are consistent with the results of the pull-down assay. Together, we conclude that the Hfq-binding ability of 3′-extended forms of sRNAs is heavily impaired.

### Transcription termination is enhanced under stress conditions in which sRNAs are induced

**FIGURE 7.**
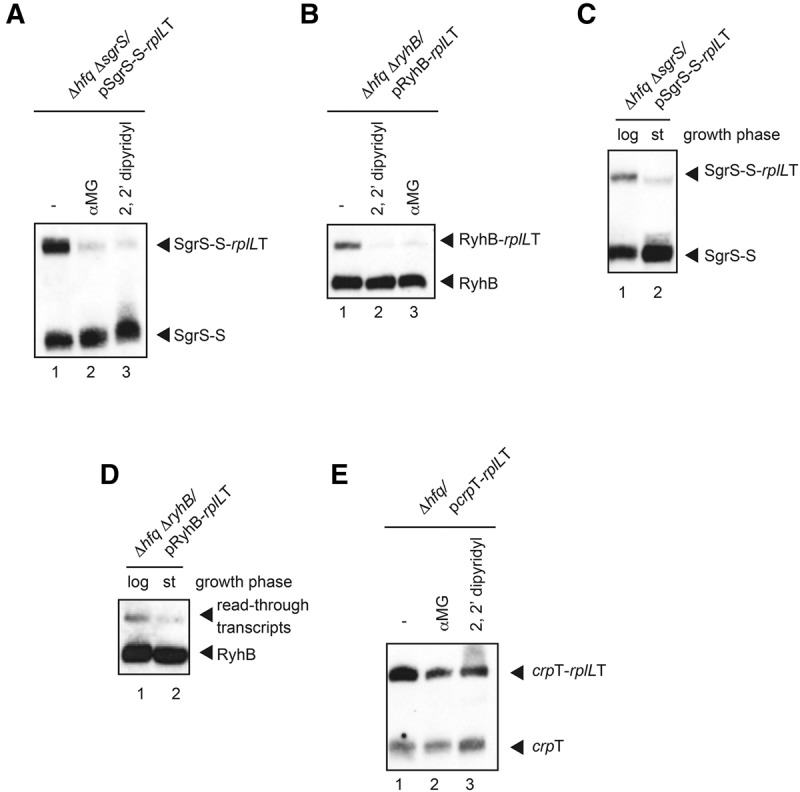
Transcriptional readthrough at the Rho-independent terminators of sRNA genes under stress conditions. (*A*) Effects of glucose-phosphate stress or Fe^2+^ depletion on readthrough at the *sgrS* terminator. TM772 (Δ*sgrS* Δ*hfq*) cells harboring pSgrS-S-*rplL*T were grown in LB medium in the presence of 0.2% arabinose. At *A*_600_ = 0.6, 0.1% α-metylglucoside (αMG) or 250 μM 2, 2′-dipyridyl (dip) was added to cultures and incubation was continued for 10 min. Total RNAs were prepared and then the RNA samples were subjected to Northern blotting using SgrS-S probe. Ten μg (lanes *1*,*2*) or 25 μg (lane *3*) of RNAs were loaded. (*B*) Effects of glucose-phosphate stress or Fe^2+^ depletion on readthrough at the *ryhB* terminator. TM820 (Δ*ryhB* Δ*hfq*) cells harboring pRyhB-*rplLT* were grown in LB medium in the presence of 1.0% arabinose. At *A*_600_ = 0.6, 250 μM dip or 0.1% αMG was added to cultures and incubation was continued for 10 min. Total RNAs were prepared and then the RNA samples were subjected to Northern blotting using the RyhB probe. Four micrograms (lanes *1*,*3*) or 10 μg (lane *2*) of RNAs were loaded. (*C*,*D*) Effect of growth phase on the readthrough at *sgrS* and *ryhB* terminators. TM772 (Δ*sgrS* Δ*hfq*) cells harboring pSgrS-S-*rplL*T (*C*) and TM820 (Δ*ryhB* Δ*hfq*) cells harboring pRyhB-*rplL*T (*D*) were grown in LB medium in the presence of 1.0% arabinose. Total RNAs were prepared from the cultures of *A*_600_ = 0.6 (log phase) and *A*_600_ = 2.5 (stationary phase). The RNA samples (10 μg) were subjected to Northern blotting using the SgrS-S (*C*) or RyhB (*D*) probes. (*E*) Effects of glucose-phosphate stress and Fe depletion on the readthrough at the *crp* terminator. TM589 (Δ*hfq*) cells harboring p*crp*T-*rplL*T were grown in LB medium in the presence of 0.2% arabinose. At *A*_600_ = 0.6, 0.1% αMG or 250 μM dip was added to cultures and incubation was continued for 10 min. Total RNAs were prepared and then the RNA samples were subjected to Northern blotting using the *crp* probe. Four micrograms (lanes *1*,*2*) or 10 μg (lane *3*) of RNAs were loaded.

So far, we have examined transcription termination at Rho-independent terminators of sRNA genes in normally growing cells. Hfq-binding sRNAs are expressed from their own genes on the chromosome under specific stress and/or physiological conditions. For example, SgrS and RyhB are induced in response to glucose-phosphate stress such as accumulation of glucose-6-phosphate and to depletion of Fe^2+^, respectively. Because transcription of sRNA genes and their activities are subject to control by Rho-independent termination, we examined the effect of stress on Rho-independent termination of sRNA genes. We first tested the effect of glucose-phosphate stress on transcription termination at the *sgrS* terminator. Cells harboring pSgrS-S-*rplL*T were grown in LB medium containing 0.2% arabinose to mid-log phase and exposed to 0.1% nonmetabolizable glucose analog α-methylglucoside (αMG) for 10 min. Then, total RNAs were prepared and subjected to Northern blotting using the *sgrS* probe. Interestingly, the addition of αMG markedly reduced the amount of the readthrough product SgrS-S-*rplL*T (Fig. 7A, lanes 1,2). In other words, the glucose-phosphate stress enhanced transcription termination at the *sgrS* terminator. We also examined the effect of Fe^2+^ depletion on transcription termination at the *sgrS* terminator. Cells harboring pSgrS-S-*rplL*T were grown in LB medium containing 0.2% arabinose to mid-log phase and treated with 2,2′-dipyridyl for 10 min to deplete Fe^2+^. The addition of 2,2′-dipyridyl clearly enhanced transcription termination at the *sgrS* terminator (Fig. 7A, lane 3). Next, we examined the effects of glucose-phosphate stress and Fe^2+^ depletion on transcription termination at *ryhB* terminator by using cells harboring pRyhB-*rplL*T. As shown in Figure 7B, termination efficiency at *ryhB* terminator was also enhanced by both glucose-phosphate stress and Fe^2+^ depletion. Thus, transcription termination at the Rho-independent terminators of sRNA genes is enhanced not only by the cognate stress but also by noncognate stress.

It is interesting to test whether other stress conditions that are associated with the induction of sRNAs affect the transcription termination at the Rho-independent terminators. It is known that several sRNAs including RybB are well expressed during stationary phase compared with log phase (Vogel et al. 2003). Therefore, we have examined the effect of growth phase on transcription termination at Rho-independent terminators. Cells harboring pSgrS-S-*rplL*T or pRyhB-*rplL*T were grown in LB medium containing 1.0% arabinose. Total RNAs were prepared from cells at mid-log and stationary phases, and subjected to Northern blotting using the *sgrS* or *ryhB* probe. As shown in Figure 7, C and D, transcription termination at Rho-independent terminators of these sRNA genes is markedly enhanced during stationary phase. Thus, it is likely that transcription termination at Rho-independent terminators of sRNAs is generally enhanced under stress conditions. Another interesting question is whether the enhanced termination specific of sRNA genes. To address this question, we have constructed the *crp*T*-rplL*T hybrid gene and tested the effect of stress on the Rho-independent terminator of *crp*. The results show that the termination at the *crp* terminator is markedly enhanced by the stress (Fig. 7E). Thus, the enhanced termination under stress conditions may be a general effect at any Rho-independent transcription terminators throughout the genome.

### Analysis of readthrough with endogenous transcripts

**FIGURE 8.**
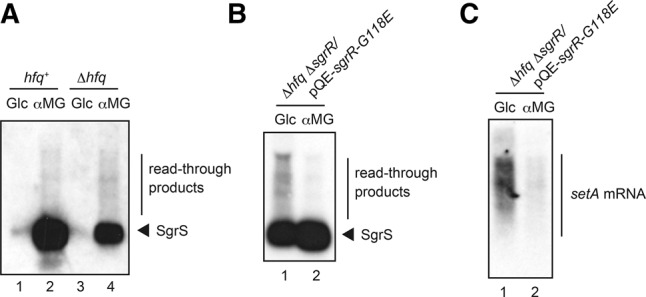
Transcriptional readthrough at the endogenous *sgrS* terminator and the effect of the stress. (*A*) IT1568 (*hfq*^+^) and TM589 (Δ*hfq*) cells were grown in LB medium. At *A*_600_ = 0.6, 0.1% glucose or αMG was added to cultures and incubation was continued for 10 min. Total RNAs were prepared and then the RNA samples (4 μg) were subjected to Northern blotting using SgrS probe. (*B*,*C*) TM816 (Δ*hfq* Δ*sgrR*) cells harboring pQE-*sgrR-G118E* were grown in LB medium in the presence of 0.1 mM IPTG. At *A*_600_ = 0.6, 0.1% glucose or αMG was added to cultures and incubation was continued for 10 min. Total RNAs were prepared and then the RNA samples (20 μg) were subjected to Northern blotting using SgrS probe (*B*) or *setA* probe (*C*), respectively.

We have demonstrated above that transcripts frequently read through the terminators of sRNA genes on the plasmid and transcription termination is enhanced under stress conditions. It is interesting to analyze the transcription termination/readthrough with the endogenous transcripts generated from chromosomal sRNA genes. For this, we have focused on the *sgrS-setA* operon. The *hfq*^+^ and *hfq*^−^ cells were grown in LB medium to mid-log phase and exposed to either 0.1% glucose or αMG for 10 min. Then, total RNAs were prepared and subjected to Northern blotting. As reported previously (Vanderpool and Gottesman 2004; Kawamoto et al. 2005), SgrS is dramatically induced by αMG (Fig. 8A, lanes 2,4). We also confirmed the previous observation (Kawamoto et al. 2005) that the level of SgrS in the *hfq*^+^ cells is much higher than that in the *hfq*^−^ cells because of the stabilization of SgrS by Hfq. In addition, the readthrough transcripts can be clearly observed as heterogeneous smear bands. Thus, the readthrough occurs with the endogenous transcripts as in the case of plasmid-derived transcripts.

It is difficult to examine the effect of stress on the readthrough at the endogenous *sgrS* terminator because the endogenous SgrS is not expressed under nonstress conditions. It is known that a mutant G118E SgrR can activate the *sgrS* transcription even without stress (Sun and Vanderpool 2011). Then, we used cells expressing this mutant SgrR to examine how the stress affects the readthrough at the endogenous *sgrS* terminator. For this, we constructed Δ*hfq* Δ*sgrR* strain (TM816) in which the C-terminal two-thirds of SgrR coding region on the genome was deleted and plasmid pQE-*sgrR-G118E* carried the mutant *sgrR* gene encoding SgrR-G118E. TM816 was transformed with pQE-*sgrR-G118E.* Cells harboring pQE-*sgrR-G118E* or pQE80L vector were grown in LB medium containing 0.1 mM Isopropyl β-d-thiogalactopyranoside (IPTG). Total RNAs were prepared from cells at mid-log phase, and subjected to Northern blotting using the *sgrS* or *setA* probe. As expected, SgrS is effectively generated in TM816 cells carrying pQE-*sgrR-G118E* independently from the stress (Fig. 8B) while it is not expressed in TM816 cells harboring the vector plasmid (data not shown). Importantly, the readthrough was markedly reduced under the glucose-phosphate stress (Fig. 8B). The readthrough and its reduction under the stress were also observed in Northern analysis using the *setA* probe (Fig. 8C). Thus, we conclude that the transcription termination at the endogenous *sgrS* terminator transcripts is enhanced under stress conditions.

## DISCUSSION

Genes encoding Hfq-binding sRNAs end with a Rho-independent transcription terminator. A striking feature of Rho-independent terminators of sRNA genes is the presence of a long consecutive T stretch (more than seven) in the nontemplate strand (Otaka et al. 2011). We showed previously that the RNA sequence corresponding to the Rho-independent transcription terminator makes up a large part of the Hfq-binding module of sRNAs (Ishikawa et al. 2012). Thus, modulation of transcription termination is important for generation of functional Hfq-binding sRNAs in cells. Major questions we have addressed in this study are: (1) how frequently transcriptional readthrough occurs at sRNA genes; (2) whether Hfq affects transcription termination at sRNA genes; (3) whether the readthrough products, the 3′-extended forms of sRNAs, retain Hfq-binding ability; and (4) how stress conditions associated with the induction of sRNAs affect transcription termination. We used a “double terminator system” in which a strong Rho-independent terminator was placed just downstream from the Rho-independent terminators of the plasmid-borne sRNA genes *sgrS* and *ryhB*.

We found first that ∼50% and 20% of elongating RNA polymerases read through the Rho-independent terminators of *sgrS* and *ryhB* during normal growth (Figs. 2B,4A). Although the stability of the terminator hairpin and the length of polyU tail are important factors that dictate the termination efficiency, there seems to be a little difference between *sgrS* and *ryhB* terminators regarding the stability and the length of polyU tail. In fact, the free energy of the terminator hairpin (ΔG) of SgrS and RyhB are calculated to be −11.3 and −10.7, respectively, while the length of the polyU tail of SgrS and RyhB are 8 and 9, respectively (Fig. 1B). It is known that several factors including flanking sequences of a terminator hairpin affect termination efficiency at Rho-independent terminators (Reynolds et al. 1992). The presence of a short hairpin before the terminator hairpin of SgrS (Ishikawa et al. 2012) may limit the formation of terminator hairpin leading to the reduced transcription termination (increased readthrough) at the *sgrS* terminator.

It is an intriguing question if Hfq plays any roles in transcription termination at the Rho-independent terminators in particular of sRNA genes because Hfq interacts with nascent transcripts of the sRNA genes. However, it has not been tested experimentally whether Hfq affects transcription termination at Rho-independent terminators until the present work. It is reported that Hfq reduces transcription termination by directly inhibiting the activity of termination factor Rho at Rho-dependent terminators (Rabhi et al. 2011). Yet, here we see that the levels of readthrough product are little or only slightly affected by the presence of Hfq at the *sgrS* and *ryhB* terminators while the abundance of terminated transcripts is elevated because these RNAs are stabilized against degradation by Hfq binding. The observation that Hfq does not affect readthrough simply implies that Hfq is not involved in modulation of transcription termination at Rho-independent terminators.

The finding that the transcriptional readthrough occurs frequently at the *sgrS* and *ryhB* terminators implies that significant amounts of readthrough products are continuously generated in cells. The products generated by readthrough are variants of sRNAs in which their 3′ ends are extended. The 3′-extended forms of SgrS and RyhB retain the entire sequence corresponding to the Hfq-binding module (Ishikawa et al. 2012) though the polyU stretch is no longer located at the 3′ end in these RNA molecules. This raised the question of whether the 3′-extended forms of SgrS and RyhB still retained their regulatory function as Hfq-binding sRNAs. Hfq is known to stabilize sRNAs upon binding in cells (Storz and Gottesman 2006). We demonstrated that the 3′-extended forms of SgrS and RyhB are poorly stabilized by Hfq (Figs. 3, 4), and by pull-down assays that the Hfq-binding ability of the 3′-extended forms of SgrS and RyhB is dramatically decreased (Fig. 5). We also showed by a competition assay using a biotin–streptavidin system that a synthetic RNA corresponding to the 3′-extended form of SgrS does not bind to Hfq in vitro (Fig. 6). We conclude that the 3′-extended forms of SgrS and RyhB produced by transcriptional readthrough no longer retain the Hfq-binding ability and thereby may not function to regulate the target mRNAs. This conclusion is consistent with the early mutational study on DsrA sRNA that showed that mutations in the terminator stem-loop of DsrA led to a significant readthrough resulting in the loss of the regulatory function of DsrA (Sledjeski and Gottesman 1995). In addition, it is reported that the affinity and/or the mode of Hfq binding to a hexauridine (U6) was significantly altered by a single nucleotide substitutions (U5A, U5C, and U5G) at the 3′-terminal position (Sauer and Weichenrieder 2011). This is also consistent with the present finding that the readthrough products of Hfq-binding sRNAs no longer bind to Hfq. Thus, we conclude that the polyU stretch must be located at the 3′ end to act as an element to bind Hfq.

Studies to understand how Hfq-binding sRNAs interact with the target mRNAs and Hfq are crucial in understanding how sRNAs act. Various biochemical and biophysical methods have been used to analyze sRNA–mRNA and/or sRNA–Hfq interactions. For example, we previously used an electrophoretic mobility shift assay to analyze the sRNA–mRNA and sRNA–Hfq interactions using RNAs prepared by either in vitro transcription or chemical synthesis (Maki et al. 2010). Another approach for studying RNA–RNA and RNA–protein interactions is the use of biotin-labeled RNAs along with streptavidin beads. Indeed, this approach has proved to be useful to study the sRNA–mRNA interaction (Vincent et al. 2013). In this study, as we have outlined in results, we used the biotin–streptavidin system to study the interaction between sRNAs and Hfq. Our system consists of a 5′-biotinylated synthetic RNA containing a minimal Hfq-binding module, a test RNA, purified Hfq and streptavidin-conjugated magnet beads. The biotinylated RNA binds tightly to the streptavidin beads without losing the Hfq-binding activity. Thus, Hfq can be immobilized on the beads through biotinylated RNA. When excess amounts of test RNAs retaining but not losing Hfq-binding activity can compete with the biotinylated RNA resulting in the inhibition of the Hfq binding to the beads. We showed that this method is quite simple, rapid, and useful to examine the Hfq-binding ability of individual RNA molecules. To our knowledge, this is the first case in which a biotinylated RNA has been successfully used to study the sRNA–Hfq interaction although sRNAs tagged by aptamers such as MS2 and boxB were shown to be useful for the in vivo study on Hfq–sRNA interaction (Said et al. 2009).

The *sgrS* gene is known to be co-transcribed with the downstream gene *setA* encoding a putative sugar transporter (Sun and Vanderpool 2011). SetA was shown to contribute to the cellular response to the glucose-phosphate stress although how it acts remains to be determined (Sun and Vanderpool 2011). Similarly, the *ryhB* seems to be co-transcribed with the downstream gene *yhhX* (Vassinova and Kozyrev 2000) though this has not been shown experimentally so far. We demonstrated in the present study that transcription termination at the Rho-independent terminators of *sgrS* and *ryhB* is markedly enhanced under the stress conditions. The enhanced termination under the stress conditions was also observed in the genomic *sgrS-setA* transcript (Fig. 8). Thus, the sRNA production is regulated not only at the step of transcription initiation but also at the step of transcription termination. This is biologically significant since increased transcription termination at sRNA genes under stress conditions generate more stable functional sRNAs to deal with the stresses. The enhanced transcription termination at sRNA genes implies that the expression of downstream genes is quite low. In fact, the analysis of the *sgrS-setA* bi-cistronic transcript showed high SgrS levels relative to *setA* mRNA levels under glucose-phosphate stress (Sun and Vanderpool 2011). According to our data, the abundance of SgrS-S-*rplL*T in *hfq*^+^ cells is a few % of that of SgrS-S under the stress conditions, indicating most transcripts terminate at the *sgrS* terminator. It remains to be determined whether this differential expression of the sRNA gene and the downstream gene has biological significance under these stress conditions. We also do not know at this stage the mechanism by which transcription termination is enhanced under stress conditions. Interestingly, the transcription termination at *sgrS* and *ryhB* terminators is enhanced not only by a particular cognate stress but also other stress conditions. This suggests that the physiology of cells undergoing stress conditions affects transcription termination at the Rho-independent terminators. One plausible effect of stress might be to reduce nucleoside triphosphates (NTPs) levels in cells, since low NTP concentrations are reported to enhance transcription termination at Rho-independent terminators in vitro (Reynolds et al. 1992). It is interesting to examine if stress conditions such as accumulation of glucose-6-phosphate and depletion of Fe^2+^ ion lead to the reduction of NTP concentration in cells, and if this is a general effect at Rho-independent transcription terminators throughout the genome.

## MATERIALS AND METHODS

### Bacterial strains and plasmids

The *E. coli* K12 strains and plasmids used in this study are listed in Table 1. IT1568 (W3110 *mlc*^−^) was used as a parent wild-type strain. To construct TM772 and TM820, the Δ*hfq*::*cat* allele of TM587 (Morita et al. 2005) was moved to TM542 (Kawamoto et al. 2005) and TM635 (Morita et al. 2006), respectively, by P1 transduction. To construct TM821, the *hfq-Flag-cat* allele of TM615 (Morita et al. 2005) was moved to TM635 (Morita et al. 2006) by P1 transduction. TM803 and TM822 were constructed by removing the *cat* gene flanked by two FRT sequences from TM771 (Otaka et al. 2011) and TM821, respectively. The Δ*sgrR::kan* allele was constructed from BW25113/pKD46 according to Datsenko–Wanner protocol using pKD4 harboring *kan* gene (Datsenko and Wanner 2000). To construct TM814, the Δ*sgrR::kan* allele was moved to IT1568 by P1 transduction. To construct TM816, the Δ*hfq*::*cat* allele of TM587 (Morita et al. 2005) was moved to TM814 by P1 transduction.

**TABLE 1.**
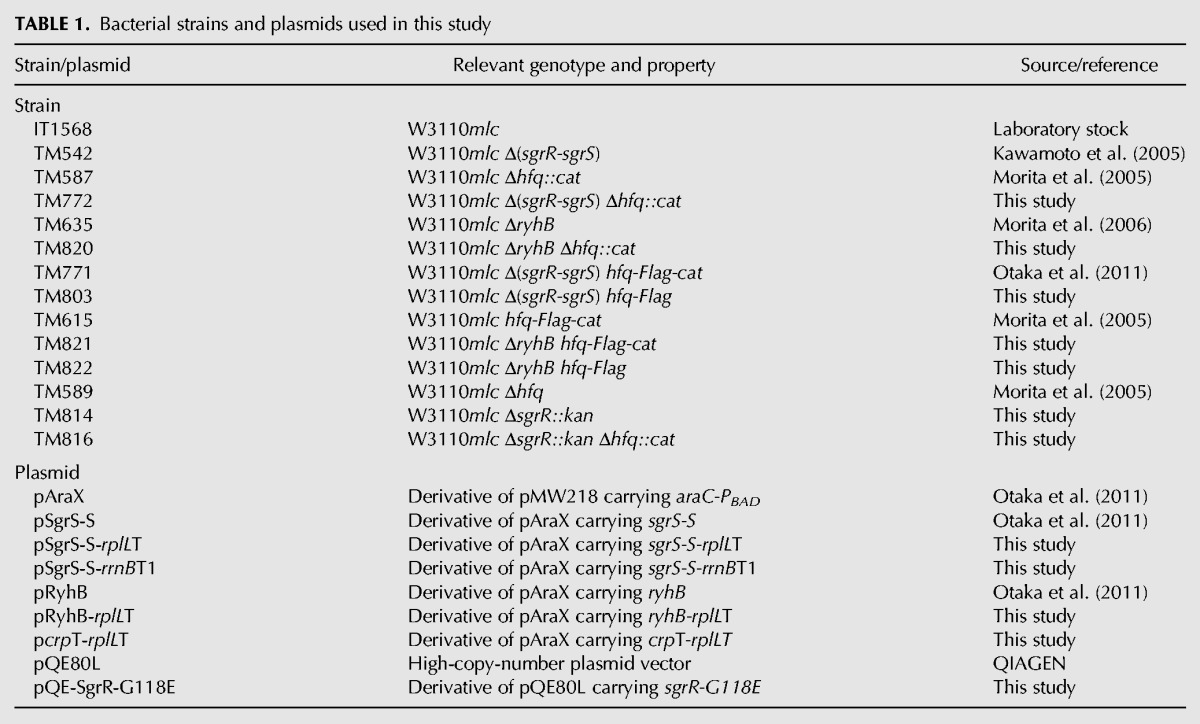
Bacterial strains and plasmids used in this study

The DNA primers used are listed in Table 2. Plasmids pSgrS-S-*rplL*T and pSgrS-S-*rrnB*T1 were constructed as follows: pSgrS-S was used to amplify the DNA fragments containing the *sgrS-S* sequence and the *rplL*T or *rrnB*T1 sequence with primers 1127 and 1415 or 1707, respectively. The amplified DNA fragments were digested with *Xba*I and *Hin*dIII and cloned into pAraX. Plasmid pRyhB-*rplL*T was constructed as follows: pRyhB was used to amplify the DNA fragment containing the *ryhB* sequence and the *rplL* terminator sequences with primers 1144 and 1527. The amplified DNA fragment was digested with *Xba*I and *Hin*dIII and cloned into pAraX. Plasmid p*crp*T-*rplL*T was constructed as follows: pHA7 (Aiba et al. 1982) was used to amplify the DNA fragment containing the *crp* terminator region and the *rplL* terminator sequence with primers 1717 and 1718. The amplified DNA fragment was digested with *Xba*I and *Hin*dIII and cloned into pAraX. Plasmid pQE-*sgrR-G118E* was constructed as follows: chromosomal DNA of W3110*mlc* was used to amplify the DNA fragment 1 containing the mutated *sgrR* region (from −19 to +366 relative to ATG start codon) with primers 1596 and 1597. Similarly, DNA fragment 2 containing the mutated *sgrR* region (+341 to +1656 relative to ATG start codon) was amplified with primers 1595 and 1598. Then, the DNA fragments 1and 2 were used to amplify the mutated *sgrR*, in which G at +353 is changed to A with 1597 and 1598. The amplified DNA fragment containing *sgrS G118E* was digested with *Eco*RI and *Hin*dIII and cloned into pQE80L.

**TABLE 2.**
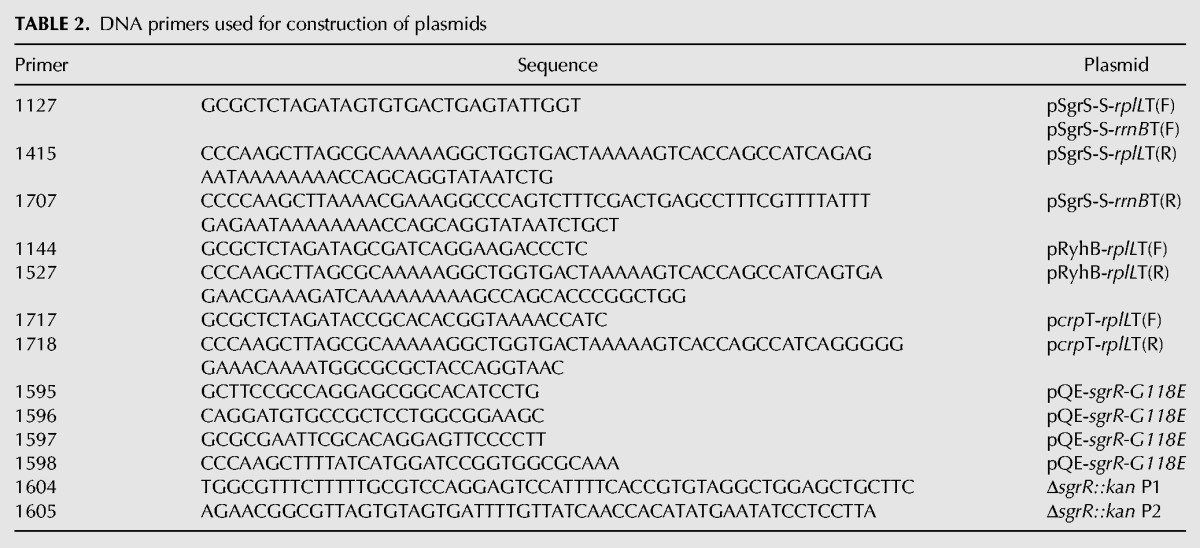
DNA primers used for construction of plasmids

### Northern blotting

Cells carrying the indicated plasmids were grown at 37°C to mid-log phase unless specified in LB medium supplemented with kanamycin (15 μg/mL) and indicated amounts of arabinose. In Figure 8B,C, cells carrying the indicated plasmids were grown at 37°C to mid-log phase with ampicillin (50 μg/mL) and indicated amounts of IPTG. Total RNAs were isolated as described (Aiba et al. 1981). To detect SgrS-S, RyhB, and *crp* RNAs, RNA samples were resolved by 12%, 6%, and 8% polyacrylamide gel electrophoresis in the presence of 8 M urea and blotted onto Hybond-N^+^ membrane (GE Healthcare). To detect *ptsG* mRNA and *setA* mRNA, RNA samples were resolved by 1.2% agrarose gel electrophoresis in the presence of formaldehyde and blotted onto Hybond-N^+^ membrane (GE Healthcare). To detect SgrS and tmRNA, RNA samples were resolved by 1.5% agrarose gel electrophoresis in the presence of formaldehyde and blotted onto Hybond-N^+^ membrane (GE Healthcare). The RNAs were visualized using digoxigenin (DIG) reagents and kits for nonradioactive nucleic acid labeling and a detection system (Roche Applied Science) according to the procedure specified by the manufacturer. The SgrS-S RNA probe corresponding to antisense of 3′ portion (+168 to +227) of *sgrS* was prepared by DIG RNA labeling kit (Roche Applied Science). The following DIG-labeled DNA probes were prepared by PCR using DIG-dUTP: a 305-bp fragment corresponding to the 5′ region of *ptsG* (*ptsG* probe); 95-bp fragment corresponding to the *ryhB* (RyhB probe); 227-bp fragment corresponding to the *sgrS* (SgrS probe); 363-bp fragment corresponding to the tmRNA (tmRNA probe); 90-bp fragment corresponding to the 3′ region of *crp* (*crp* probe); 220-bp fragment corresponding to +11 to +230 region relative to ATG start codon of *setA* (*setA* probe). Multi Gauge ver. 3.1 software (Fujifilm) was used to quantify RNA bands on the films.

### Pull-down assay

Cells were grown in 400 mL of LB medium in the presence of 1.0% arabinose, harvested, and washed with 10 mL STE buffer (100 mM NaCl, 10 mM Tris–HCl at pH 8.0, and 1 mM EDTA). The cell pellets were suspended in ice cold 10 mL of IP buffer (20 mM Tris–HCl at pH 8.0, 0.1 M KCl, 5 mM MgCl_2_, 10% glycerol, and 0.1% Tween20). The cell suspension was sonicated and centrifuged at 10,000*g* for 10 min at 4°C. The supernatant (crude extract: CE) was incubated with 50 μL of anti-Flag M2-agarose suspension (Sigma-Aldrich) for 30 min at 4°C. The mixture was filtered by using a mini chromatography column (Bio-Rad). The agarose beads were washed twice by 10 mL of IP buffer. The proteins bound to the beads were eluted with 50 μL of IP buffer containing 0.4 mg/mL Flag peptide (Sigma-Aldrich) and used as bound fraction (B). To analyze proteins, the crude extract (10 μL) and the bound fraction (2 μL) were mixed with SDS-PAGE loading buffer (6.25 mM Tris–HCl at pH 6.8, 2% SDS, 10% glycerol, 5% *b*-mercaptoethanol, 0.1% Bromophenol blue). The samples were heated for 5 min at 100°C and subjected to a 15% SDS gel electrophoresis, and then transferred to an Immobilon membrane (Milipore). The membrane was treated with an anti-Flag monoclonal antibody (Sigma-Aldrich). Signals were visualized by the Lumi-light Western Blotting Substrate (Roche). To analyze RNAs, the crude extract (10 μL) and the bound fraction (2 μL) were treated with phenol, precipitated, and washed with ethanol. Each precipitate was dissolved in 6 μL of RNA buffer (0.02 M sodium acetate at pH 5.5, 0.5% SDS, and 1 mM EDTA). The RNA samples were subjected to Northern blotting.

### Purification of His-tagged Hfq

TM589 harboring pQE80L-Hfq-His (Kawamoto et al. 2006) was cultured in 200 mL of LB medium at 37°C in the presence of 25 μM IPTG and 50 μg/mL ampicillin. At *A*_600_ = 0.2, 2 mM of IPTG was added to the culture and incubation was continued for 70 min. Cells were harvested and washed with 20 mL STE, and suspended in Lysis Buffer Native (pH 8.0) (Qiagen). The cell suspension was treated with lysozyme (1 mg/mL) for 10 min at 0°C, sonicated, and centrifuged at 10,000*g* for 10 min at 4°C. The supernatant was treated with RNase A (1 μg/mL) for 10 min at 0°C and then heated for 10 min at 80°C. The sample was centrifuged at 10,000*g* for 10 min at 4°C. The supernatant was incubated with 500 μL of Ni^2+^-NTA agarose resin (Qiagen) for 20 min at 4°C and Hfq-His_6_ protein was purified according to the manufacturer's instruction. Purified Hfq-His_6_ was concentrated by Amicon Ultra (0.5 mL 3K) Centrifugal Filters (Milipore). The Hfq-His_6_ concentration was estimated by 15% SDS-PAGE and SimplyBlue SageStain (Invitrogen). Purified Hfq-His_6_ was stored with storage buffer (20 mM Tris–HCl at pH 8.0, 0.1 M KCl, 5 mM MgCl_2_, 50% glycerol, and 0.1% Tween20, 1 mM DTT) at −30°C.

### In vitro competition assay

Synthetic RNAs including 5′- biotinylated RNA were obtained from GeneDesign Inc. Two microliters of Dynabeads MyOne Streptavidin C1 magnetic beads (Lifetechnologies), purified Hfq-His_6_ (66.7 nM), indicated amounts of biotinylated RNA, and an excess amount (667 nM) of different competitor RNAs were mixed together in 15 μL of binding buffer (20 mM Tris–HCl at pH 8.0, 1 mM DTT, 1 mM MgCl_2_, 20 mM KCl, 10 mM Na_2_HPO_4_–NaH_2_PO_4_ at pH 8.0) containing 1 mg of yeast tRNA (Invitrogen Japan). The mixtures were gently shaken for 10 min at 37°C. Streptavidin magnetic beads were collected by magnet, and 10 μL of supernatant was used as unbound fraction (UB). Streptavidin magnetic beads were washed with 100 μL of wash buffer (20 mM Tris–HCl at pH 8.0, 0.2 M KCl). Ten microliters of H_2_O was added to streptavidin magnetic beads, and the suspension was used as bound fraction (B). The UB and B samples were mixed with SDS-PAGE loading buffer. The sample was heated for 5 min at 100°C and subjected to a 15% SDS gel electrophoresis. Hfq-His_6_ was visualized by SilverQuest silver staining kit (Life Technologies).
